# Role in aromatic metabolites biodegradation and adverse implication of denitrifying microbiota in kitchen waste composting

**DOI:** 10.1186/s40793-023-00496-8

**Published:** 2023-05-30

**Authors:** Mingzi Shi, Caihong Song, Lina Xie, Guogang Zhang, Zimin Wei

**Affiliations:** 1 Tianjin Key Laboratory of Animal and Plant Resistance, College of Life Sciences, Tianjin Normal University, Tianjin, 300387 China; 2 College of Life Science, Liaocheng University, Liaocheng, 252000 China; 3 College of Life Science, Henan Agricultural University, Zhengzhou, 450000 China

**Keywords:** Denitrifying microbiota, Biodegradation, Kitchen waste composting, Aromatic metabolites, Biochar

## Abstract

**Background:**

Understanding the functional diversity, composition, and dynamics of microbiome is critical for quality in composting. Denitrifying microbiota, possessing multiple metabolic pathways simultaneously. Denitrification-based biodegradation of aromatic metabolites has been widely applied in the bioremediation of sediments. However, role in biodegradation of denitrifying microbiota in kitchen waste composting remain unclear. In this study, microbiome and metabolome were used to comprehensively decipher the relationship of denitrifying microbiota and aromatic metabolites, and its implication in kitchen waste (KW) composting.

**Results:**

This study was investigated by adjusting moisture content 60% as control test (CK), 70% as denitrification test (DE). In addition, one tests referred as DE + C, which received 10% of biochar to amend denitrification. Results indicated the quantities of denitrification genes *narG* were 1.22 × 10^8^ copies/g in DE at the 55th day, which were significantly higher than that in CK and DE + C (*P* < 0.05). Similarly, the abundance of *nirK* gene also significantly increased in DE (*P* < 0.05). The relative abundance of denitrification-related microbes in DE was higher than that in CK, DE + C could weaken their abundance. Metabolomics results demonstrated that metabolites were downgraded in aromatic amino acid and catechin metabolic pathways in DE, which were identified as precursors to synthesis key product fulvic acid. The concentrations of fulvic acid dramatically decreased 21.05 mg/g in DE comparison with CK. Biochar addition alleviated the biodegradation of aromatic metabolites and reduced the utilization of fulvic acid. Integrative analyses of metabolomics and microbiome suggested that the microbiota involved in nitrite reduction pathway was vital for the biodegradation aromatic metabolites. Mantel test verified that NO_3_^-^-N, moisture content, eta, environmental factors were important drivers behind the changes in the denitrifying microbiota biodegradation function.

**Conclusion:**

The data confirm the biodegradation function of denitrifying microbiota led to the loss of core product fulvic acid in KW composting, which highlighted the adverse role and implication of denitrification for composting humification. Control of denitrification with biochar was recommended to improve composting quality.

**Supplementary Information:**

The online version contains supplementary material available at 10.1186/s40793-023-00496-8.

## Introduction

Kitchen waste (KW) is defined as residue from food processing, catering service, residential activities [1]. Densely populated areas generate tons of KW daily [2]. KW contains plentiful organic molecules and extremely high moisture, and can be converted into new resources if properly treated and processed [2, 3]. However, KW entering the mixed-municipal waste system is tight to deal with by conventional methods, such as landfill and incineration [4]. Composting is an economically viable method for treating KW, converting it into high-quality soil amendments or organic fertilizers [3]. KW composting were characterized by the severe anaerobic nature, because aerobic microbes consume oxygen faster through respiration than replenish it through diffusion [5]. In addition, although sufficient moisture content is essential for microbial growth and organic matter mineralization, the abundant moisture content in KW accelerates the anaerobic conditions [6]. This is due to the insolubility of oxygen in water, resulting in decreased the permeability and fluidity of air during composting. Therefore, plentiful nutrient and high moisture make KW composting a desirable environment for microbial anaerobic biodegradation [7].

Biodegradation of under denitrification conditions, a process mediated by denitrifying microbiota to gradual decompose aromatic rings, utilizing nitrate as electron acceptor and nitrogen source as well as aromatic compounds as electron donor and carbon source [8, 9]. Denitrification-based biodegradation of polycyclic aromatic hydrocarbons has been applied in the bioremediation of aromatic hydrocarbon-contaminated sediments [9–11]. Currently, many denitrifying bacteria have proven the ability to degrade aromatic compounds [12, 13]. Rabus et al. presented the complete genome of representative multi-functional denitrifying bacteria EbN1 strain. Ten anaerobic aromatic degradation pathways and four aerobic aromatic degradation pathways were characterized in EbN1 strain, indicating that were capable to degrade aromatic compounds under both aerobic and anaerobic conditions [14, 15]. KW composting not only contained high level of nutrients and moisture, but also presented heterogeneous and porous structures, resulting in aerobic and anoxic environments at the same time. Therefore, KW composting provides predominant conditions for denitrifying microbiota-based biodegradation of aromatic compounds. However, the biodegradation of aromatic compounds under denitrification conditions in KW composting remained to be elucidated, which hindered the comprehensive understanding of the composting humification.

As the typical aromatic compounds in composting, the level of humic substances is the key indicator for humification [16]. Humic substances consist of humic acid, fulvic acid and humin [17, 18]. Fulvic acid present relatively simple structure and is more easily used by microbes [16]. In addition, as the main component of humic substances in KW composting, fulvic acid are aromatic macromolecular substances with several hypothetical formation pathways, including polyphenol theory, lignin theory and Maillard reaction [16, 19, 20]. All these hypothetical pathways are involved in precursor polymerization and condensation [16, 20, 21]. It is generally believed that precursors of fulvic acid are aromatic compounds with low-molecular, such as phenols, quinones and aromatic amino acids [16, 21]. However, under denitrification conditions, the interaction between these aromatic precursors and fulvic acid has not been reported. This study combined metabolomics to detect these aromatic metabolites, and comprehensively decipher the relationship of denitrifying microbiota and aromatic metabolites.

In this study, the biodegradation of aromatic compounds under denitrification conditions in KW composting was elucidated. Firstly, denitrifying-related microbiota were identified and analyzed. Low-molecular aromatic metabolites were measured via GC-MS and basic aromatic metabolism pathways were profiled. Secondly, the biodegradation of denitrifying microbiota was elucidated and the crucial aromatic precursors of fulvic acid were identified. Finally, this study integrated the metabolomics with microbiome data to explore the potential cause and driver for denitrifying microbiota-based biodegradation of aromatic metabolites. In addition, a strategy was proposed to alleviated the biodegradation of denitrifying microbiota. The research provided a reverse thinking into understanding humification of KW composting and was important for improving composting quality.

## Methods

### Sample collection and experimental design

KW were collected from Tiedong Street vegetable market (Harbin, China). The main effective composition of KW for composting were vegetables and fruits. The KW was further sorted by hand and all plastic and other non-degradable contaminants removed. Table S1 listed the detailed initial properties of KW. The specifications of the reactor and the detailed of composting technology can be found in previous research [22]. Sawdust were was used for the adjustment of carbon/nitrogen ratio and mixed with KW thoroughly. The mixture was divided equally into nine (included parallel test groups) composting reactors. Three groups of tests were set up and noted as experiments control test (CK) and denitrification test (DE), and denitrification biochar test (DE + C). Since moisture content is a key factor affecting denitrification. Plentiful moisture makes KW composting a desirable environment for denitrification. The biodegradation of aromatic compounds based on denitrification was investigated by adjusting moisture content in different composting tests. Specifically, adjusted the initial moisture content of approximately 60% as CK, 70% as DE. Furthermore, biochar could mitigate N_2_O emission and microbial denitrification [23]. Therefore, one tests received an additional 10% of biochar amendment and were referred as DE + C, to investigative the effect of denitrification regulation on aromatic compounds biodegradation. The details of tests were listed in Table S2. The duration of the composting tests was 55 days. Based on the previous research, intermittent aeration with a rate of 0.5 L kg^− 1^ min^− 1^ was employed [22]. There were no further adjustments to the moisture content throughout the composting period.

### Determination of chemical analysis

The KW composting sampling was performed on days 2, 5, 9, 15, 25, and 55 according to the quartile. Moisture content (MC) was determined by drying method. Temperatures were recorded daily with a digital thermometer. Organic matter (OM) was assayed by burning method. Total carbon (TC) and total nitrogen (TN) content were measured by elemental analyzer. Total phosphorus (TP) and available phosphorus (AP) was determined using the ascorbic acid/molybdate reagent color method [24]. pH was tested with calibrated electrodes. NO_3_^−^-N, NH_4_^+^-N and NO_2_^−^-N were extracted with 2.0 M KCl [25, 26], and were assayed using colorimetry and spectrophotometry. N_2_ was analyzed by a high-precision nitrogen analyzer. Aromatic compounds fulvic acid was extracted by NaOH and Na_4_P_2_O_7_ mixed solution based on previous research [27]. Fulvic acid concentration was characterized by total organic carbon, measured using a TOC-Vcph analyzer. All tests were performed in triplicate.

### DNA extraction

Composting samples were firstly treated with decaying buffer and PBS to avoid humic substances interference on DNA extraction. According to manufacturer’s instruction of Omega Soil DNA Kit, genomic DNA were purified from composting samples and eluted by 100 µL 70 °C preheated Elution Buffer. Extracted DNA samples were quantified and stored at -20 °C. In addition, the qualities of DNA samples were ensured by measuring OD_260 nm_ and OD_280 nm_, with the ratio ranging between 1.8 and 2.0 [28].

### Quantification of functional genes

qPCR was executed using AceQ® Universal SYBR qPCR Master Mix (Vazyme). Estimation of the abundances of denitrification functional (*narG*, *nirS*, *nosZ*) and aromatic compounds (phenylalanine) degrading functional genes (*paaK*) by qPCR were determined successfully [29, 30]. Specific primers and reaction conditions were listed in Table S3 and Table S4. In brief, 10 µL real-time PCR reactions were proceeded with ABI 7500 thermocycler (Life Technologies, USA). The fluorescence signals were collected at extension steps of each cycle on the FAM channel [31, 32]. Positive result was determined If Ct value was less than 35 and the amplification curve was S-shaped. Negative result was determined if Ct value was reported as undetermined and fluorescent signal remained at background level. The gene copy numbers were calculated according to the formula: Y (copies/µL) = [DNA concentration X (ng/µL) × 10^− 9^/DNA fragment length (bp) × 660] × 6.02 × 10^23^ [31, 32].

### Microbiome analysis

Different tests samples at heating phase, thermophilic phase and maturity phase performed *16*S rRNA high-throughput sequencing. V3-V4 regions of composting samples were amplified using the primers 341F (5’-CCTAYGGGRBGCASCAG-3′) and 806R (5’-GGACTACNNGGGTATCTAAT-3′) [33, 34]. PCR products were mixed in equal volume and purified by GeneJET Kit. Sequencing library was constructed with Ion Plus Fragment Library Kit 48 rxns and quality assessment was performed on the Qubit@ 2.0 Fluorometer, followed by library sequencing on an Ion S5 TM XL platform. Sequences analysis were then used for OTUs (Operational taxonomic units) analysis. [33, 34].

### Metabolite analysis

The metabolites were determined by gas chromatography-mass spectrometry (GC-MS). 500 mg samples were accurately weighed, followed by grinding, centrifugation, freeze-drying, re-dissolution and volatilization. Methoxylamine hydrochloride in pyridine solution was used for oximation, followed by adding the derivatization reagent, n-hexane and internal standard in turn. Samples were then stored at injection bottles for GC-MS analysis [35, 36].

The program of GC-MS mainly included DB-5MS capillary column. The temperature of injection port was 260 ^o^C and the injection volume was 1.0 µL. Initial temperature of column maitained at 60 ^o^C for 0.5 min. The gradient heating program was 8 ^o^C / min from 60 ^o^C to 125 ^o^C and kept for 5 min, 8 ^o^C / min from 125 ^o^C to 210 ^o^C and kept for 5 min, 15 ^o^C / min from 210 ^o^C to 270 ^o^C and kept for 5 min, 20 ^o^C / min from 270 ^o^C to 305 ^o^C and kept for 5 min. The obtained GC/MS raw data was analyzed with MS-DIAL and metabolite characterization was based on LUG database [35, 36].

### Statistical analyses

Data were analyzed using Origin 2021 and SPSS statistical software. The bacteria taxa at genus level significantly correlated with denitrification genes (*P* < 0.05), were selected based on the Pearson correlation coefficient [37]. Co-occurrence network visualized denitrification-related microbiota [37]. Log2 fold change was performed between-group comparisons (DE vs. CK and DE vs. DE + C) of metabolites and microbial abundance. Procrustes analysis and Mantel test were conducted to analyze the relationships between physical-chemical parameters and denitrification-related microbes [38].

## Results and discussion

### Differences of denitrification potential

**Fig. 1:**
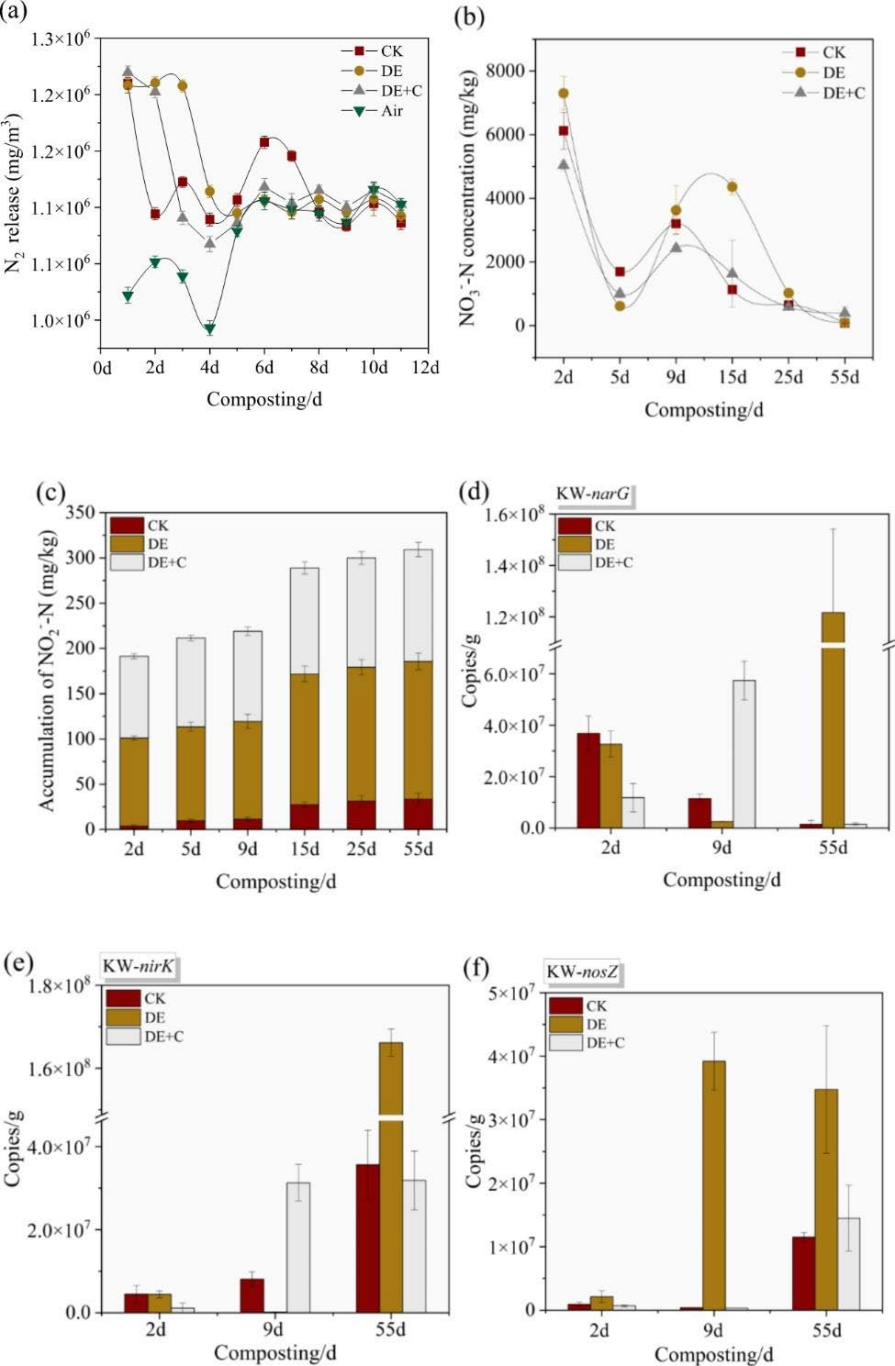
Differences of denitrification performance. (a) N_2_ release; (b) NO_3_^−^-N concentration; (c) accumulation of NO_2_^−^-N; (d) the copies number of *narG* gene; (e) the copies number of *nirK* gene; (f) the copies number of *nosZ* gene

The analysis of denitrification-related physicochemical indicators shown in Fig. 1. N_2_ as denitrification end product was mainly concentrated during heating period, and peaked at the 1st day in all tests (*P* > 0.05) (Fig. 1a). At the 2nd day, the release of N_2_ significantly decreased to 1.09 × 10^6^ mg/m^3^ in CK, but it reached 1.21 × 10^6^ mg/m^3^ and 1.20 × 10^6^ mg/m^3^ in DE and DE + C respectively. The release of N_2_ in DE remained at a high level of 1.21 × 10^6^ mg/m^3^ at the 3rd day, significantly higher than CK and DE + C (*P* < 0.05). It indicated that denitrification aggravated N_2_ release. Biochar mitigated nitrogen losses due to denitrification. The denitrification substrate NO_3_^−^-N concentration showed a downward trend during composting, indicating that the nitrate reduction reaction occurred in all tests (Fig. 1b). The initial NO_3_^−^-N concentration was 7.30 × 10^3^ mg/kg in DE, but it reduced to 6.12 × 10^3^ mg/kg, and 5.04 × 10^3^ mg/kg in CK, and DE + C. The final NO_3_^−^-N concentration was no significant difference among the three tests (*P* > 0.05). It demonstrated that DE accelerated the consumption of NO_3_^−^-N. Denitrification performance was further characterized by NO_2_^−^-N accumulation. NO_2_^−^-N was an intermediate product of the reaction. The results showed that NO_2_^−^-N accumulation was much higher in DE than CK and DE + C. It was in the range from 33.49 mg/kg (CK) to 152.24 mg/kg (DE) (Fig. 1c). These results indicated that DE had an excellent denitrification potential. In addition, biochar addition could mitigate denitrification performance.

The *narG*, *nirS* and *nosZ* genes are involved in nitrate reduction, nitrite reduction and nitrous oxide reduction pathways, respectively. They are generally used as the molecular markers of denitrifying microbes [39, 40]. It was indicated from Fig. 1 (d, e, f) that the quantities of the *narG*, *nirK* genes in composting were about three folds higher than *nosZ* genes. In addition, denitrification genes were most prevalent during maturity phase (55 days). However, there were significant differences in the abundance of denitrification function genes among the tests (*P* < 0.05). The quantities of *narG* were 1.22 × 10^8^ copies/g in DE at the 55th day, which were significantly higher than that in CK and DE + C (*P* < 0.05) (Fig. 1d). Similarly, the abundance of *nirK* gene also significantly increased in DE (*P* < 0.05) (Fig. 1e). The quantities of the *nirK* gene in DE were 4.65, 5.22 folds higher than that of CK and DE + C at the 55th day, respectively. Subsequently, the abundance of *nosZ* gene in DE peaked at 3.92 × 10^7^ copies/g during thermophilic phase (9 days). At the 55th day, the abundance of *nosZ* gene was still maintained at a high level in DE (3.48 × 10^7^ copies/g), whereas it was remarkably decreased to 1.15 × 10^7^ copies/g and 1.45 × 10^7^copies/g in CK and DE + C (Fig. 1f). These results proved that the intensive of nitrate reduction, nitrite reduction and nitrous oxide reduction existed in DE. The DE + C caused decline of *narG*, *nirS* and *nosZ* genes, indicating that biochar inhibited the denitrification.

### Understanding of changes in denitrifying-related microbiota

**Fig. 2:**
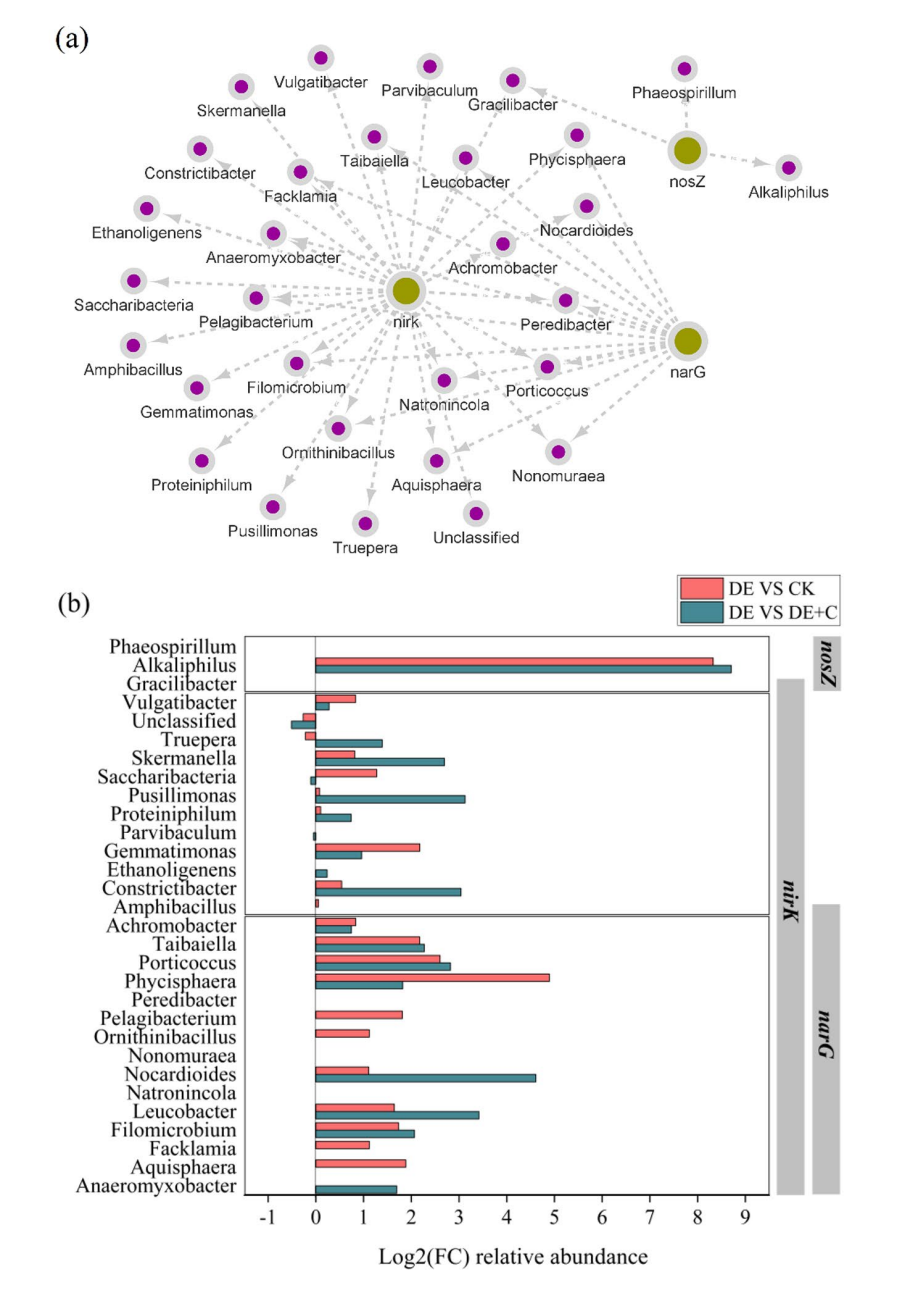
Changes of denitrifying-related microbiota. (a) the microbes related to *narG*, *nirK*, and *nosZ* genes. Purple circles represented microbes, and yellow circles represented genes. Dashed arrows indicated significant positive relationships (*P* < 0.05); (b) the Log2 fold change of denitrifying-related microbiota in CK, DE + C compared with DE

Co-occurrence network was used to visually display the genera that showed significant positive correlation with denitrification functional genes (*P* < 0.05). As shown in Fig. 2a, there were 14, 28, and 3 of microbes related to *narG*, *nirK*, and *nosZ* genes, respectively, indicating that the microbes mediating nitrite reduction were rich in KW composting. Notably, 13 microbes, including *Taibaiella*, *porticoccus*, *Ornithinibacillus*, etc., were significantly positively correlated with both *narG* and *nirK* (*P* < 0.05), suggesting that these microbes could participate in nitrate and nitrite reduction. Similarly, *Gracilibacter* could participate in nitrite and nitrous oxide reduction.

The relative abundance differential distribution of denitrifying-related microbiota was further investigated from all tests. Log2 fold change (Log2 FC) of microbes in CK, DE + C compared with DE were shown in Fig. 2b. The results found that almost all denitrification-related microbes were more abundant in DE. *Natronincola*, *Nonomuraea*, *Peredibacter*, *Gracilibacter*, and *Phaeospirillum* were the specific denitrification-related microbes in DE. Several microbes, including *Alkaliphilus* (DE VS CK: Log2 fold change = 8.33; DE VS DE + C: Log2 fold change = 8.70), *Taibaiella* (DE VS CK: Log2 fold change = 2.18; DE VS DE + C: Log2 fold change = 2.27), *Porticoccus* (DE VS CK: Log2 fold change = 2.60; DE VS DE + C: Log2 fold change = 2.82), *Phycisphaera* (DE VS CK: Log2 fold change = 4.89; DE VS DE + C: Log2 fold change = 1.82), were obviously enriched in DE compared to CK and DE + C (Fig. 2b). The above results indicated that denitrification could improve the relative abundance of related microbes, while the biochar addition could weaken their abundance. The potential ability of denitrifying-related bacteria with high abundance in DE to degrade aromatic metabolites would be further explored.

### Alterations of aromatic metabolites

**Fig. 3:**
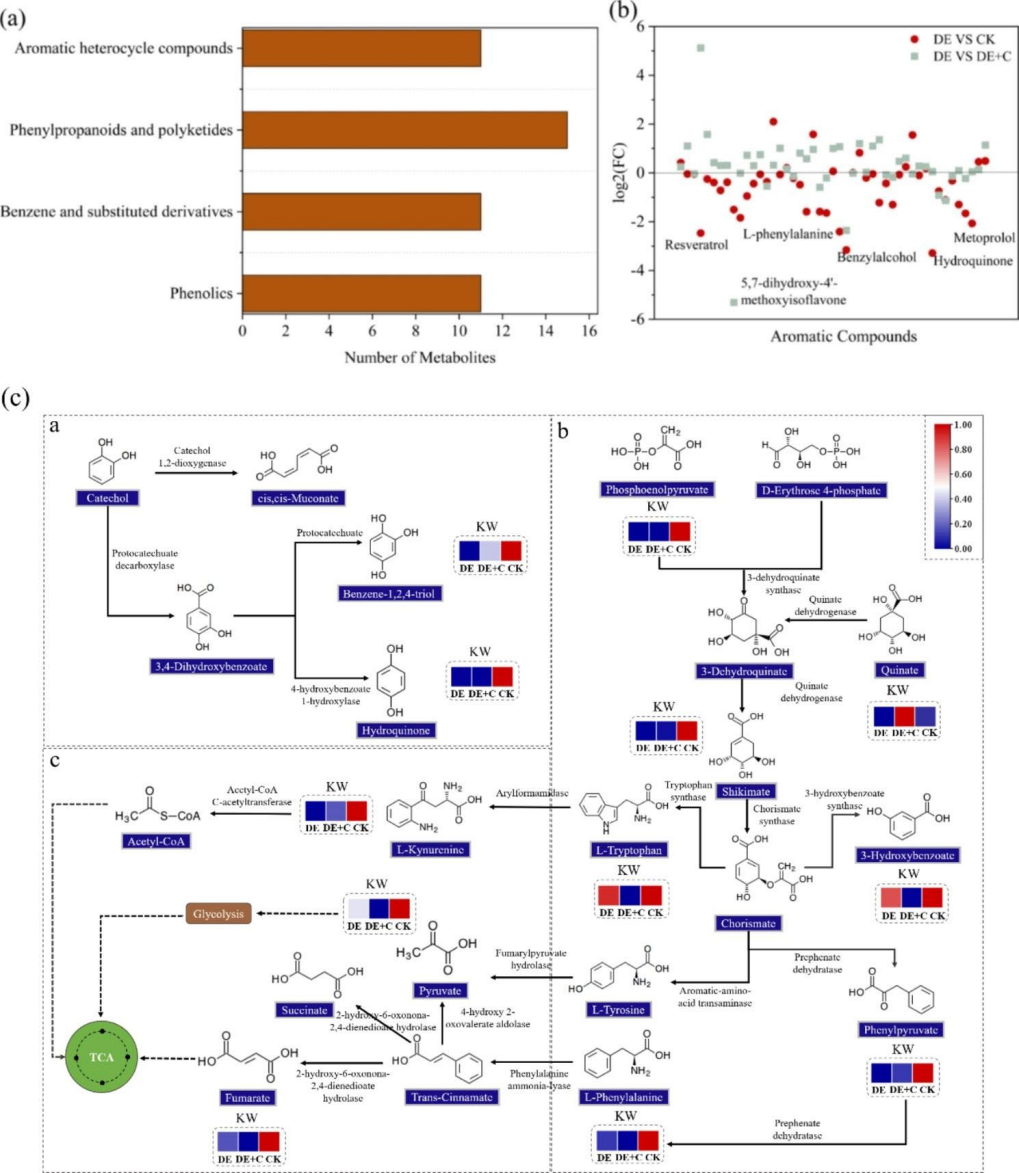
Characteristics of aromatic compound metabolism. (a) the classification of aromatic compounds metabolites; (b) Log2 fold change of aromatic compounds metabolites in CK, DE + C compared with DE; (c) Schematic diagram of the aromatic metabolic pathways during KW composting

For explored the biodegradation under denitrification, 49 of aromatic metabolites were measured via GC-MS, including phenolics (11), benzene and substituted derivatives (11), phenylpropanoids and polyketides (15), aromatic heterocycle compounds (11) (Fig. 3a). The results of comparing the Log2 fold change of aromatic compounds in different tests showed that denitrification caused obviously difference on aromatic metabolite contents. In comparison with the CK and DE + C, DE were characterized by the decrease of aromatic compounds, for example, 5,7-dihydroxy-4’-methoxyisoflavone (DE vs. CK: Log2 fold change = -1.50; DE vs. CK: Log2 fold change =-5.31) and benzylalcohol (DE vs. CK: Log2 fold change = -3.16; DE vs. CK: Log2 fold change =-2.36) (Fig. 2b).

Further pathway analysis of metabolites showed that catechin and aromatic amino acids metabolic pathway were more active in KW composting (Fig. 3c). In the catechin metabolic pathway, benzene-1,2,4-triol and hydroquinone were synthesized, which often as precursor of aromatic humic substances [41], were decreased in DE. DE + C mitigated the magnitude of the decline. Phenylalanine and tryptophan belonged to aromatic amino acids, which were synthesized by shikimate metabolism pathway [42], were decreased in DE. Aromatic metabolites phosphoenolpyruvate, 3-dehydroquinate, 3-hydroxybenzoate, phenylpyruvate were down-regulated in DE compared with CK and DE + C. Phenylalanine and tryptophan were further catabolized to pyruvate and fumarate. Result showed that pyruvate and fumarate were also decreased in DE. It might be due to decreased levels of metabolic substrates. These results suggested that denitrification robustly promoted the degradation of aromatic compounds. In particular, biochar addition could regulate denitrification and alleviate the degradation of aromatic compounds.

### Identification of aromatic metabolites as crucial precursors of fulvic acid

**Fig. 4:**
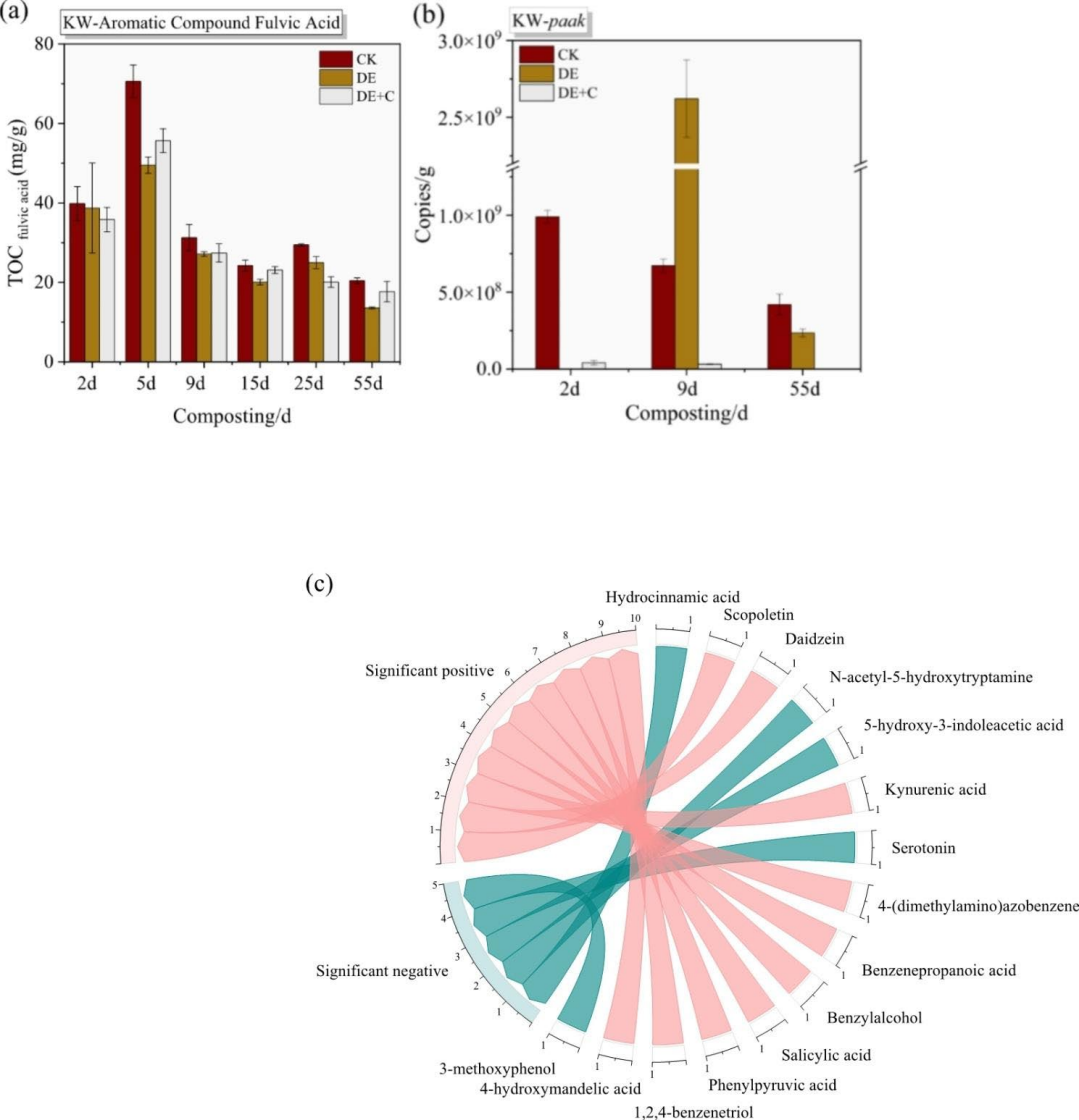
Effect of denitrification on fulvic acid. (a) the concentration of fulvic acid; (b) the copies number of aromatic-degrading functional gene *paak*; (c) the aromatic metabolites significantly related to fulvic acid (*P* < 0.05)

In comparison with other solid organic wastes, KW composting were characterized by the enrichment of available fulvic acid. Figure 4a illustrated the effect of denitrification on aromatic compounds fulvic acid. Results showed that the fulvic acid concentration reached 70.60 mg/g in CK, and it decreased to 49.55 mg/g in DE at the 5th day. It indicated that partial fulvic acid was utilized in DE. In addition, fulvic acid concentration was 55.66 g/kg at the 5th day in DE + C, indicating that the exogenous addition of biochar reduced the utilization. And the similar phenomenon was also observed during maturity phase. Thus, denitrification promoted the degradation of key aromatic compound of composting.

The possible aromatic metabolites precursors of fulvic acid were identified by correlating (Fig. 4c). It was found that scopoletin, daidzein, kynurenic acid, 4-(dimethylamino) azobenzene, benzenepropanoic acid, benzylalcohol, salicylic acid, phenylpyruvic acid, 1,2,4-benzenetriol, 4-hydroxymandelic acid was significantly positively correlated with fulvic acid (*P* < 0.05). In addition, hydrocinnamic acid, N-acetyl-5-hydroxytryptamine, 5-hydroxy-3-indoleacetic acid, serotonin, 3-methoxyphenol showed a significant negative correlation with fulvic acid (*P* < 0.05). The results suggested that the aromatic metabolites were inextricably linked to the formation of fulvic acid. These aromatic metabolites might act as precursors to synthesis fulvic acid macromolecules through specific biological and non-biological pathways.

To further clarify the effect of the degradation of denitrification on aromatic compounds, the aromatic-degrading functional gene *paak* was quantified (Fig. 4b). *paak* gene encoded phenylacetate-CoA ligase, which was involved in the degradation pathway of aromatic amino acid phenylalanine [43]. As shown in Fig. 4b, the copy numbers of *paak* genes in DE were peaked at 2.62 × 10^9^, which were 3.90 and 5.22 folds higher than that in CK and DE + C at the 9th day. It indicated that denitrification might stimulated aromatic-degrading functional gene elevation, which was the underlying reason for denitrification promoted the degradation of aromatic compounds. However, *paak* gene only encoded phenylacetate-CoA ligase. There were still many genes involved in the degradation of aromatic compounds that need to be further excavated and verified.

### Interactions among metabolites and denitrifying microbiota

**Fig. 5:**
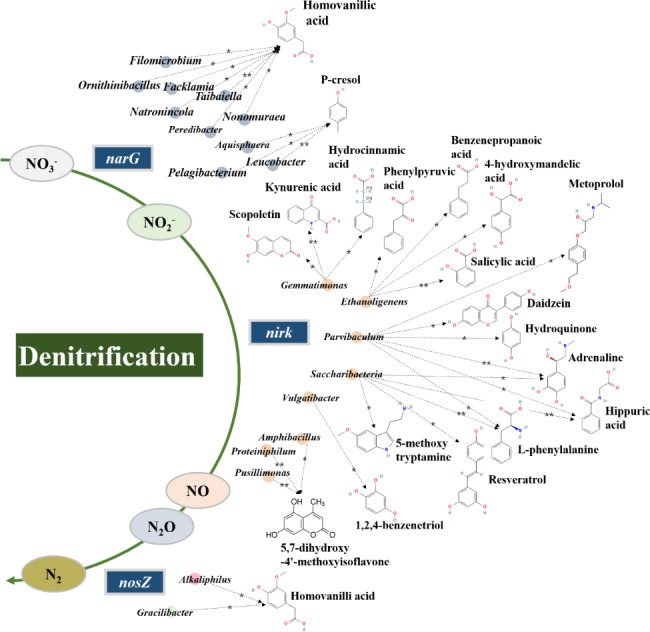
Network relationship diagram of aromatic metabolites and denitrifying-related microbiota. The blue, yellow, pink, green circles represented microbes that were significantly positively correlated with both *narG* and *nirK*, *nirK*, both *nirk* and *nosZ*, *nosZ*, respectively. Dashed arrows indicated significant negative relationships. ** meant *P* < 0.01; * meant *P* < 0.05

Considering that the generation and transformation of metabolites were associated with microbial activities. Therefore, to understand the potential interplay between obviously altered aromatic metabolites and differentially abundant denitrifying-related microbiota, association analysis using network inference based on Spearman correlation (Fig. 5). The results revealed that aromatic metabolites were negatively correlated with most denitrifying-related microbiota. Among the 10 microbes involved in both nitrate and nitrite reduction pathways, *Natronincola*, *Facklamia*, *Taibaiella*, *Ornithinibacillus*, *Nonomuraea*, *Filomicrobium*, *Peredibacter* was significantly negative correlated with aromatic metabolite homovanillic acid (*P* < 0.05). *Leucobacter*, *Pelagibacterium*, *Aquisphaera* were significantly associated with p-cresol (*P* < 0.05). Among the 8 microbes involved in nitrite reduction pathway, the association between *Vulgatibacter* and 1,2,4-benzenetriol was significant negative (*P* < 0.05). *Parvibaculum* was significantly negative correlated with 6 aromatic metabolites, including hydroquinone, adrenaline, L-phenylalanine, hippuric acid, daidzein, metoprolol (*P* < 0.05). Similarly, *Saccharibacteria* was significantly negative correlated with adrenaline, L-phenylalanine, 5-methoxytryptamine, resveratrol, hippuric acid (*P* < 0.05). *Ethanoligenens* and *Gemmatimonas* had significantly negative correlations with benzenepropanoic acid, salicylic acid, phenylpyruvic acid, 4-hydroxymandelic acid and hydrocinnamic acid, kynurenic acid, scopoletin, respectively (*P* < 0.05). *Amphibacillus*, *Proteiniphilum*, *Pusillimonas* was significantly negative correlated with 5,7-dihydroxy-4’-methoxyisoflavone (*P* < 0.05). In addition, nitrous oxide reducing bacteria *Alkaliphilus* and *Gracilibacter* significantly showed negative correlation with Homovanillic acid (*P* < 0.05). Taken together, there were multitudinous significantly negative associations among aromatic metabolites and denitrifying-related microbiota, suggesting that these taxa might participate in the degradation of aromatic compounds. Moreover, from the identification results of aromatic metabolites as crucial precursors of fulvic acid (Fig. 4c), aromatic metabolites hydrocinnamic acid, scopoletin, daidzein, 1,2,4-benzenetriol, benzenepropanoic acid, salicylic acid, phenylpyruvic acid, 4-hydroxymandelic acid were identified as potential precursors of fulvic acid, indicating that denitrifying-related microbiota also played a crucial role in the reduction of fulvic acid formation. It was noteworthy that the distribution of associations was different across the denitrification pathway. The network relationships comprised more negative associations between aromatic metabolites and denitrifying-related microbiota during nitrite reduction. It indicated that microbiota involved in nitrite reduction pathway was vital for the biodegradation aromatic metabolites in KW composting.

### Analysis of key drivers altering denitrifying microbiota metabolic function

**Fig. 6:**
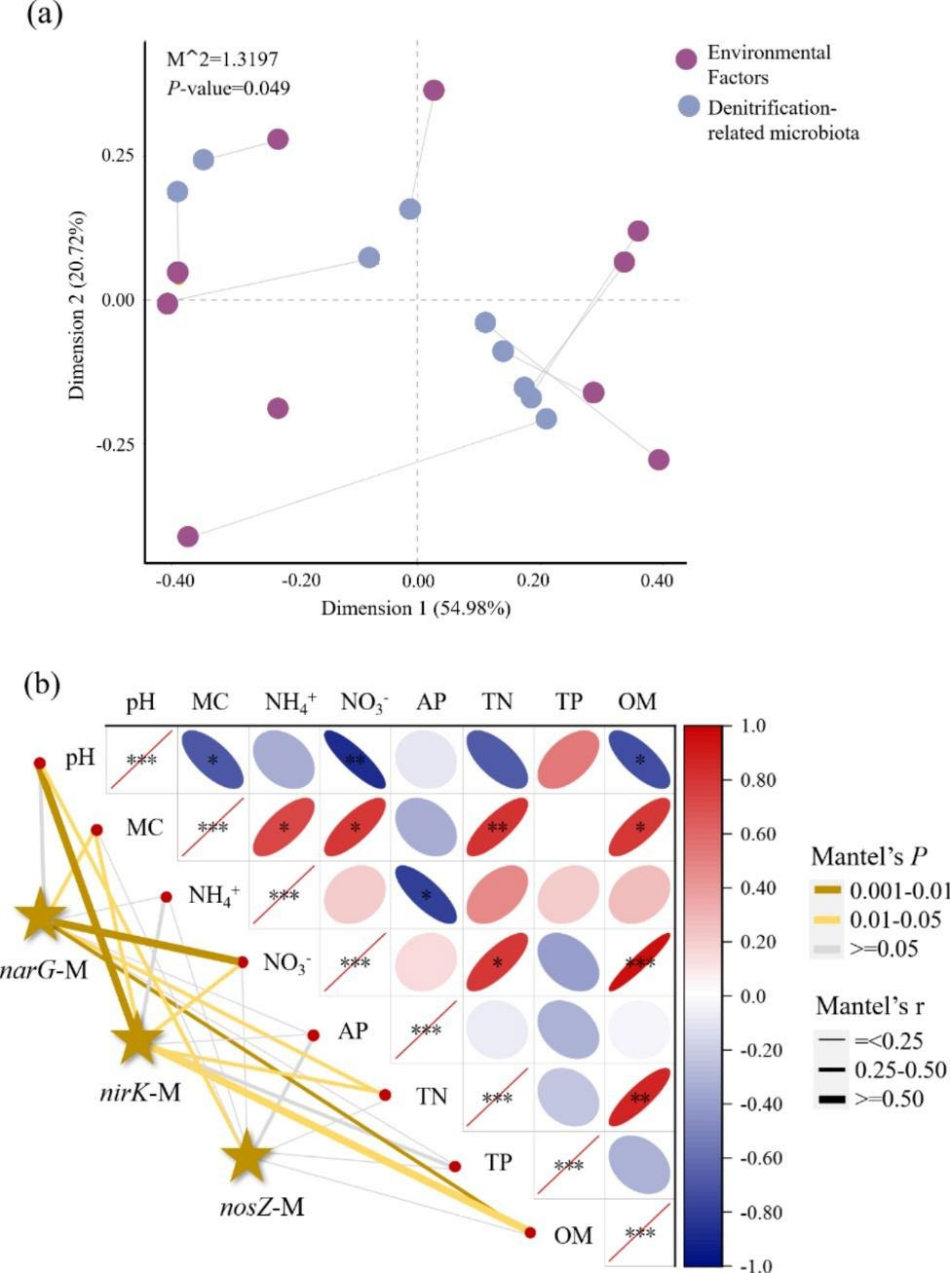
The influence of environmental factors on denitrifying -related microbes using the Procrustes analysis (a) and Mantel test (b). *narG*-M, *nirK*-M, *nosZ*-M, represented microbes associated with *narG*, *nirK*, *nosZ*, respectively. *** meant *P* < 0.001; ** meant *P* < 0.01; * meant *P* < 0.05

For determining the key driving force behind the changes in the denitrifying microbiota metabolic function, Procrustes analysis of the link between denitrifying-related microbiota microbes and environmental factors (pH, MC, NO_3_^−^-N, NH_4_^+^-N AP, TN, TP, OM) was established. The correlation was observed to be significantly (M^2^ = 1.3198, *P* < 0.05) between denitrifying-related microbiota and environmental factors in different samples (Fig. 6a). Mantel test further revealed that the key environmental drivers jointly altered the metabolic function of denitrifying microbiota involving nitrate reduction, nitrite reduction, and nitrous oxide reduction (Fig. 6b). The denitrification electron acceptor NO_3_^−^-N showed significant (*P* < 0.05) correlations with microbes related to *narG* and *nirK*, suggesting that NO_3_^−^-N was one of the main driving factors. OM and TN had a strong (*P* < 0.05) correlation with microbes related to *narG* and *nirK*. It explained that denitrifying microbiota was restricted by nutrients. In addition, denitrifying microbiota microbes at all stages were significantly affected by MC (*P* < 0.05), confirming that excessive MC easily led to local anaerobicity and promoted denitrification. Microbes related to *nirK* and *nosZ* were more sensitive to pH, and there were significantly correlations (*P* < 0.05). It has been reported that pH was an important factor affecting the denitrification process, and the optimum pH range for denitrifying microbiota was 7.0–8.0 [44]. Meanwhile, there were also inseparable links between environmental factors. For example, MC had a significant positive correlation with OM, TN, and NO_3_^−^-N, NH_4_^+^-N (*P* < 0.05), suggesting that the differences of denitrifying microbiota metabolic function caused by multifactor interactions. Therefore, it was suggested to put forward strategies, such as controlling NO_3_^−^-N content, reducing MC, to weaken denitrification and promote humification during KW composting.

## Conclusion

In conclusion, this study revealed the high abundance of denitrifying genes and microbiota in moisture-rich KW composting. The data elucidated the biodegradation of denitrifying microbiota on aromatic metabolites, which identified as precursors of key product fulvic acid, suggesting that denitrifying microbiota play an adverse role for composting humification. Biochar addition regulated denitrifying microbiota and alleviated the biodegradation of aromatic metabolites. In addition, it was recommended to weaken denitrification by optimizing the microenvironment, which was important for improving composting quality.

## Electronic supplementary material

Below is the link to the electronic supplementary material.

Additional file 1: Table S1. Physical and chemical properties of composting raw materials, Table S3. PCR amplification primer, Table S4. PCR amplification condition, Figure S1. Changes in temperature of composting reactor, Figure S2. Agarose gel electrophoresis of PCR products for functional genes.

## Data Availability

Data is available in the additional files.
