# Supplementary material for: Role in aromatic metabolites biodegradation and adverse implication of denitrifying microbiota in kitchen waste composting
Source: Environ Microbiome. 2023 May 30;18:44. doi: 10.1186/s40793-023-00496-8 (PMC10230690; doi:10.1186/s40793-023-00496-8)
Supplement: Supplementary file 1 — Additional file 1: Table S1. Physical and chemical properties of composting raw materials, Table S3. PCR amplification primer, Table S4. PCR amplification condition, Figure S1. Changes in temperature of composting reactor, Figure S2. Agarose gel electrophoresis of PCR products for functional genes. [file 40793_2023_496_MOESM1_ESM.docx]

**Supplementary Information**

**Role in aromatic metabolites biodegradation and adverse implication of** **denitrifying microbiota in kitchen waste composting**

Mingzi Shi ^a, c^, Caihong Song ^b^, Lina Xie ^a^, Guogang Zhang ^a^, Zimin Wei ^a,^ *

^a^ Tianjin Key Laboratory of Animal and Plant Resistance, College of Life Sciences, Tianjin Normal University, Tianjin 300387, China.

^b^ College of Life Science, Liaocheng University, Liaocheng 252000, China.

^c^ College of Life Science, Henan Agricultural University, Zhengzhou 450000, China.

*Corresponding Authors:

Tianjin Key Laboratory of Animal and Plant Resistance, College of Life Sciences, Tianjin Normal University, Tianjin 300387, China

Tel/Fax: +86-018745724658

E-mail address: [weizimin@neau.edu.cn](mailto:weizimin@neau.edu.cn) (Z. Wei)

Supporting Information Includes:

- 4 tables
- 2 figures

Table S1. Physical and chemical properties of composting raw materials

| Materials | Moisture content  (%) | | Total carbon  (%) | Total nitrogen  (%) | Particle size  (mm) |
| --- | --- | --- | --- | --- | --- |
| Kitchen waste | | 85.26 ± 2.72 | 43.63 ± 2.21 | 2.78 ± 0.43 | 26.70 ± 1.43 |
| Sawdust | | --- | 63.20 ± 2.87 | 0.56 ± 0.42 | --- |

Table S2. Experimental group of composting

| Tests | Moisture content (%) | Biochar (%) |
| --- | --- | --- |
| CK  DE  DE+C | 60.45 ± 0.95 %  70.12 ± 0.78 %  70.01 ± 1.23 % | ---  ---  10% |

Table S3. PCR amplification primer

| Gene | Primer | Nucleotide sequence | Target fragment | |
| --- | --- | --- | --- | --- |
| *narG*  *nirK*  *nosZ*  *paak* | narGF  narGR  nirKF  nirKR  nirKF  nirKR  paaKF  paaKR | 5’-TAYGTSGGGCAGGARAAACTG-3’  5’-CGTAGAAGAAGCTGGTGCTGTT-3’  5’-ATCATGGTSCTGCCGCG-3’  5’-GCCTCGATCAGRTTGTGGTT-3’  5’-CCCGCTGCACACCRCCTTCGA-3’  5’-CGTCGCCSGAGATGTCGATCA-3’  5’-TACGGCTACGGCCTGTTCA-3’  5’-CTCCGGGTAGAAGTGGTC-3’ | | 110 bp  473 bp  300 bp  300 bp |

Table S4. PCR amplification condition

| Denitrifying gene | Amplification conditions |
| --- | --- |
| *narG*  *nirK*  *nosZ*  *paak* | Pre-denaturation 95°C, 3 min; denaturation 95°C, 10 s; annealing 57°C, 30 s; extension 72°C, 20 s (35 cycle); 72°C, 10 min; 4°C, 10 min.  Pre-denaturation 95°C, 3 min; denaturation 95°C, 10 s; annealing 57°C, 30s ; extension 72°C, 20 s（35 cycle）; 72°C, 10 min; 4°C, 10 min.  Pre-denaturation 95°C, 3 min; denaturation 95°C, 10 s; annealing 57°C, 30s ; extension 72°C, 20 s（35 cycle）; 72°C, 10 min; 4°C, 10 min.  Pre-denaturation 95°C, 3 min; denaturation 95°C, 10 s; annealing 50°C, 30s ; extension 72°C, 20 s（35 cycle）; 72°C, 10 min; 4°C, 10 min. |


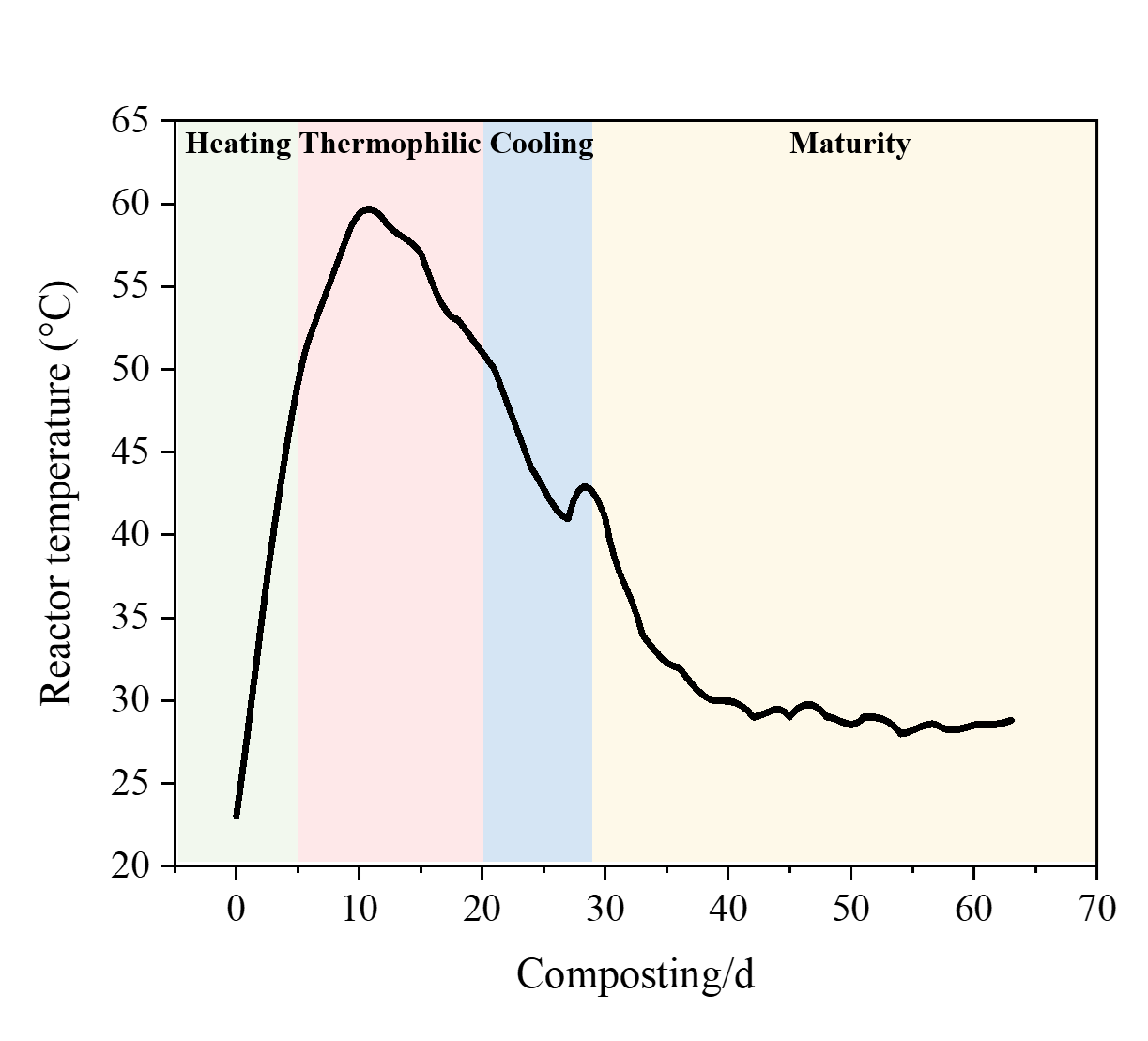


Figure S1. Changes in temperature of composting reactor.


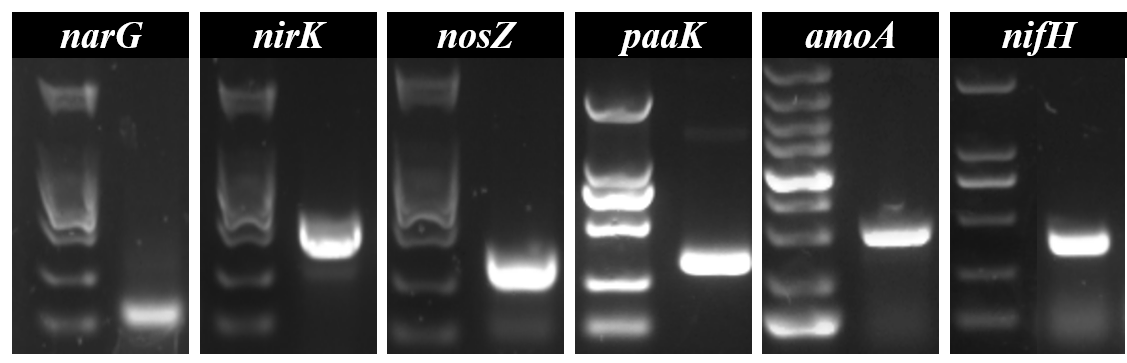


Figure S2 Agarose gel electrophoresis of PCR products for functional genes
